# Quantitative Characterization of the Growth of *Deinococcus geothermalis* DSM-11302: Effect of Inoculum Size, Growth Medium and Culture Conditions

**DOI:** 10.3390/microorganisms3030441

**Published:** 2015-08-20

**Authors:** Julie Bornot, Carole Molina-Jouve, Jean-Louis Uribelarrea, Nathalie Gorret

**Affiliations:** 1 Université de Toulouse, Institut National des Sciences Appliquées (INSA), Université Paul Sabatier (UPS), Institut National Polytechnique (INP); Laboratoire d’Ingénierie des Systèmes Biologiques et des Procédés (LISBP), 135 Avenue de Rangueil, Toulouse F-31077, France; E-Mails: julie.bornot@insa-toulouse.fr (J.B.); jouve@insa-toulouse.fr (C.M.-J.); uribelar@insa-toulouse.fr (J.-L.U.); 2 Institut National de la Recherche Agronomique (INRA), Unité Mixte de Recherche (UMR) 792 Ingénierie des Systèmes Biologiques et des Procédés, Toulouse F-31400, France; 3 Centre National de la Recherche Scientifique (CNRS), UMR5504, Toulouse F-31400, France

**Keywords:** *Deinococcus geothermalis*, strain variability, physiology, growth performances

## Abstract

Due to their remarkable resistance to extreme conditions, *Deinococcaceae* strains are of great interest to biotechnological prospects. However, the physiology of the extremophile strain *Deinococcus geothermalis* has scarcely been studied and is not well understood. The physiological behaviour was then studied in well-controlled conditions in flask and bioreactor cultures. The growth of *D. geothermalis* type strains was compared. Among the strains tested, the strain from the German Collection of Microorganisms (Deutsche Sammlung von Mikroorganismen DSM) DSM-11302 was found to give the highest biomass concentration and growth rate: in a complex medium with glucose, the growth rate reached 0.75 h^−1^ at 45 °C. Yeast extract concentration in the medium had significant constitutive and catalytic effects. Furthermore, the results showed that the physiological descriptors were not affected by the inoculum preparation steps. A batch culture of *D. geothermalis* DSM-11302 on defined medium was carried out: cells grew exponentially with a maximal growth rate of 0.28 h^−1^ and *D. geothermalis* DSM-11302 biomass reached 1.4 g·L^−1^ in 20 h. Then, 1.4 g_DryCellWeight_ of biomass (X) was obtained from 5.6 g glucose (Glc) consumed as carbon source, corresponding to a yield of 0.3 Cmol_X_·Cmol_Glc_^−1^; cell specific oxygen uptake and carbon dioxide production rates reached 216 and 226 mmol.Cmol_X_^−1^·h^−1^, respectively, and the respiratory quotient (QR) value varied from 1.1 to 1.7. This is the first time that kinetic parameters and yields are reported for *D. geothermalis* DSM-11302 grown on a mineral medium in well-controlled batch culture.

## 1. Introduction

Bacteria belonging to the *Deinococcaceae* family possess very interesting properties as they are well-known for being the most radiation-resistant bacteria. *Deinococcus* species can survive and repair their genome even after an exposition to high radiation levels [1,2] or other environmental stress agents including ultraviolet light, oxidative stress and desiccation [3,4,5]. Their ability to tolerate high temperatures, a broad range of pH and chemicals compounds make *Deinococcaceae* good candidates for production of bioenergy products such as bioethanol and metabolites derivatives [6]. To implement biological processes involving *Deinococcus* strains, a better understanding of their physiology in a bioreactor culture on a minimal medium is required. The most studied strains of *Deinococcaceae* are the mesophile one, *Deinococcus radiodurans* and the thermophilic one, *Deinococcus geothermalis*.

Many genetic engineering studies have been conducted in order to understand *Deinococcus radiodurans’* efficient system for repairing DNA, and to develop strategies for bioremediation using radiation resistant microorganisms [7,8,9].

Concerning *Deinococcus geothermalis*, it is a gram-positive microorganism of 1.5–2.0 µm in diameter which divides as tetrads and forms orange-pigmented colonies when cultivated on agar plates [2]. *D. geothermalis* was first isolated in hot springs in Naples, Italy and in São Pedro do Sul, Portugal [2]. Since its discovery, it has been identified in other locations like industrial printing paper machines where it forms coloured biofilms [10,11,12]. Due to its extreme resistance properties and its ability to grow at high temperature, *D. geothermalis* is involved in the bioremediation process of environmental radioactive waste sites and is well-studied for its solvent-tolerance and solvent-utilization as a carbon source [13]. However, very few data on its physiology and growth conditions are available. Few defined media have been described for the culture of *D. geothermalis* strains [2,13,14], and yeast extract was obviously necessary to avoid growth limitation [13]. Consequently, *D. geothermalis* is commonly cultivated in rich media containing at least one complex nutrient source such as yeast extract, peptone or tryptone [2,14]. Only two works reported high cell density production of *Deinococcaceae* strains, e.g., *Deinococcus radiodurans* and *Deinococcus geothermalis*. The cultures were performed in a 20 L fermentor with a medium containing yeast extract [15,16]. Although previous works have shown that *Deinococcaceae* have complex nutritional requirements [2,13,17,18], no study has been reported yet on the influence of the inoculum preparation on their growth properties (growth rate, maximal biomass concentration, glucose uptake). Inoculum preparation, including preservation method, revivification step and inoculum stages, is an essential element for the quality of the microbial culture and for bioprocess performances [19]. To achieve high performance culture, e.g., high biomass concentration and growth rate, a reproducible physiological state in the inoculum and a correct inoculation cell density are essential [20]. Inoculum preparation should maximize the cell viability from stock culture, provide a genotypically identical population to the one that was stored, increase biomass concentration and provide a physiological state suitable for growth in the final bioreactor [21]. A comprehensive characterization of the growth behaviour is still missing. Obviously, need arises to better quantify and understand the physiology of *Deinococcus geothermalis* and to manage all the steps from inoculum preparation up to large scale industrial production.

The aim of the present investigation was first to compare the culture of three strains of *D. geothermalis* and choose the best candidate for further physiological studies. Then, this study focused on the impact of storage conditions, revivification methods, subcultures in complex media and inoculum size on the biological activity of the strain *D. geothermalis* DSM-11302. Finally, a batch culture in a 1 L-bioreactor using a defined media with glucose as carbon source was performed to quantify *D. geothermalis* DSM-11302 physiology in well-controlled conditions. An objective of this work was also to report the first quantitative description of the dynamic behavior of a *D. geothermalis* strain grown in a bioreactor under non-limiting conditions (exponential growth) on a mineral medium.

Such a characterization is relevant in the field of bioprocess engineering as the species is a potential production host for next generation biofuels.

## 2. Materials and Methods

### 2.1. Microorganisms, Media and Growth Conditions

#### 2.1.1. Bacterial Strains

Three strains of *Deinococcus geothermalis* were used in this study. Two of them, *D. geothermalis* DSM-11301 and *D. geothermalis* DSM-11302, were received from the German Collection of Microorganisms and Cell Cultures DSMZ (Deutsche Sammlung von Mikroorganismen und Zellkulturen GmbH, Braunschweig, Germany). The strain *D. geothermalis* DSM-11300 was provided by Deinol partners (CapAlfa, Montpellier, France). The strains were inoculated into Yeast extract Peptone Dextrose (YPD) medium broth (yeast extract 5 g·L^−1^, peptone 10 g·L^−1^, dextrose 10 g·L^−1^) and incubated at 45 °C for 24 h. The bacterial cells were then stored at −80 °C with 20% (v/v) glycerol.

For the comparison of the strain preservation methods, a freeze-dried stock of *D. geothermalis* DSM-11300 received from DSMZ and stored at 4 °C was used.

#### 2.1.2. Media and Growth Conditions

For the reference inoculum preparation, the strain was plated on YPD agar medium (Yeast extract Peptone Dextrose Agar abbreviated YPDA: YPD medium broth supplemented with agar 14 g·L^−1^) for 48 h at 45 °C.

Precultures of *D. geothermalis* were carried out in a 5 mL tube containing 1.5 mL of Complex Medium Glucose (CMG) at 45 °C for 24 h on an orbital shaker (110 rpm). The Complex Medium Glucose was prepared by adding 10 g·L^−1^ of glucose, 5 g·L^−1^ of yeast extract (YE) and 2 g·L^−1^ of bacto-peptone to the mineral medium (see below). Only one colony was chosen for each inoculum. To test revivification methods, assays were made by removing the step of culture on agar plate and inoculating directly the stock suspension in liquid medium (CMG) in tubes.

For the 150 mL flask experiments, another step of pre-culture in 15 mL CMG medium was carried out, in baffled Erlenmeyer flasks with an inoculum at 10% (v/v). The flasks were incubated 12 h at 45 °C and 110 rpm. The pellet was rinsed once with physiological saline solution (9 g·L^−1^ NaCl) before inoculation.

For the bioreactor experiment, a 100 mL Defined Medium Glucose (DMG) preculture was used. This 100 mL preculture was inoculated with a 10 mL CMG preculture.

The mineral medium called Defined Medium (DM) was prepared as follows: 5.74 mM K_2_HPO_4_, 10% v/v 3-(*N*-morpholino)propanesulfonic acid (MOPS) buffer mixture (400 mM MOPS , 200 mM NH_4_Cl, 100 mM NaOH, 100 mM KOH, 2.76 mM Na_2_SO_4_, 5.28 mM MgCl_2_ and 5 µM CaCl_2_), 1% v/v of a 2 mM FeCl_3_ solution in 2 mM sodium citrate, 0.01% v/v of a micronutrients solution (3 × 10^−5^ M (NH_4_)_6_Mo_7_O_24_, 4 × 10^−3^ M H_3_BO_3_, 3 × 10^−4^ M CoCl_2_, 1 × 10^−4^ M CuSO_4_, 25 × 10^−4^ M MnCl_2_ and 10 × 10^−5^ M ZnSO_4_) and 0.01% v/v of each vitamin (niacin, thiamine hydrochloride, pyridoxine hydrochloride, cobalamin and biotin prepared separately at a concentration of 10 mg·L^−1^). The MOPS buffer mixture and the solutions of iron, micronutrients and vitamins added were sterilized by filtration. The initial pH was adjusted to 6.8 with NH_4_OH. This mineral medium DM was adapted from a medium developed for the growth of *Deinococcus radiodurans* [17] and was provided by Deinol partners. The DMG was the medium DM with 10 g·L^−1^ glucose as carbon source.

Two others media were used to compare the growth of the strains: the original medium described by Holland *et al.* [17] and the DSMZ medium 878 (*Thermus* 162 medium).

The carbon source solutions were prepared and autoclaved separately and added to the media just before the inoculation.

#### 2.1.3. Erlenmeyer Flask Experiments

##### 2.1.3.1. Culture of the Three Strains of *Deinococcus geothermalis*

Growth of the three type strains of *D. geothermalis* was evaluated using the number of generations and the glucose uptake in Erlenmeyer flask cultures. Before the experiment, 15 mL of sterile medium (DMG, CMG, *Thermus* 162 medium (TH162) or Holland medium) were added into sterile 100 mL Erlenmeyer flasks. The media were inoculated with 1.5 mL of a 12 h preculture which had been grown in CMG medium at 45 °C.

##### 2.1.3.2. Effect of Yeast Extract Concentration

**Figure A1:**
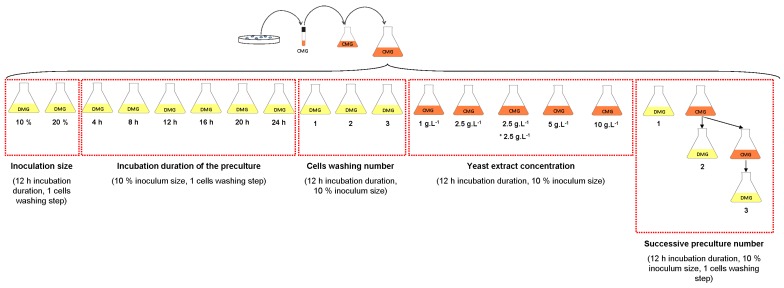
Description of the protocol for the experiments on yeast extract concentration, inoculation size, incubation duration of the preculture, cell washing number and number of precultures, in baffled Erlenmeyer flasks.

Five flasks with 150 mL CMG medium containing 1 g·L^−1^, 2.5 g·L^−1^ (two flasks), 5 g·L^−1^ and 10 g·L^−1^ of yeast extract were inoculated with 15 mL of a 150 mL CMG (5 g·L^−1^ YE) preculture. In one flask of CMG with 2.5 g·L^−1^ YE, a supplementary pulse of 2.5 g·L^−1^ YE was made when the culture reached the stationary phase (Figure A1).

Effect of the Strain Preservation Methods, the Frozen Storage Duration, the Type of Strain Revivification and the Variability between Colony Forming Units (CFU) On Petri Dishes

**Figure A2:**
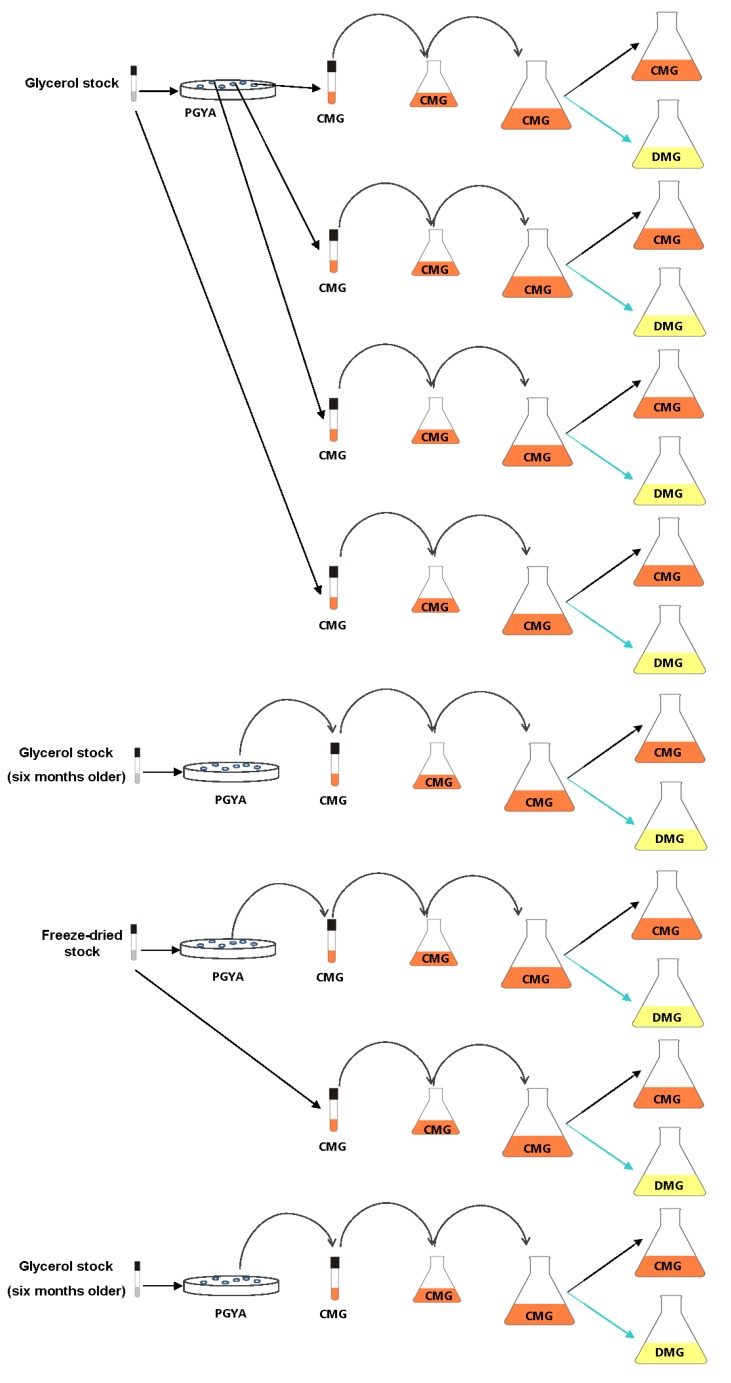
Description of the protocol for the experiments on strain preservation, frozen storage period, type of strain revivification and variability between CFU on Petri dishes, in baffled Erlenmeyer flasks (→ inoculation without cells washing step; → inoculation with one cells washing step).

The growth results in the complex medium CMG and in the defined medium DMG were compared. 150 mL of culture medium in 1 L Erlenmeyer flask were inoculated with 15 mL of a 12 h preculture in CMG, after one step of cell washing for the DMG inoculation. Freeze-dried stock and glycerol stock were compared. With the freeze-dried stock, two methods of revivification were carried out: a step of culture on agar medium (YPDA) and a direct inoculation of the stock in the liquid medium CMG in tube. With the frozen glycerol stock, these two methods of revivification were employed too. In addition, three colonies were picked on agar slants and inoculated separately in CMG broth to study the variability of growth between the colonies chosen on a solid medium. Another glycerol stock, six months older than the previous, was tested using the revivification on YPDA medium. This resulted in 7 preservation/revivification conditions and the cultures were carried out in complex and defined media (Figure A2).

##### 2.1.3.3. Effect of Inoculation Size and Incubation Duration of the Preculture

Six Erlenmeyer flasks with 150 mL DMG were inoculated at 10% (v/v) with the same CMG preculture at different incubation time: 4 h, 8 h, 12 h, 16 h, 20 h and 24 h. The inoculation size was tested by inoculating 150 mL DMG in 1 L flasks at 10% (v/v) or 20% (v/v) with a 12 h preculture in CMG (Figure A1).

##### 2.1.3.4. Effect of One- to Three-Stage Inoculum Cultures in Complex Medium

Three successive precultures of 12 h were made in 150 mL CMG. With each preculture, 150 mL DMG in 1 L flask was inoculated (Figure A1).

##### 2.1.3.5. Effect of Dilution of the Residual Yeast Extract in the Medium with Cell Washing Before Inoculation

The effect of dilution of the residual yeast extract in the inoculum, linked to the cell washing step, was studied by inoculating 150 mL DMG in 1 L Erlenmeyer flasks with cells which were washed one to three times with physiological saline solution. The preculture was a 150 mL CMG culture incubated 12 h at 45 °C (Figure A1).

##### 2.1.3.6. Growth of *Deinococcus geothermalis* DSM-11302 in Defined Media on Carbon Sources and Complex Nutrients

Fourteen media formulations were tested. The vitamins added corresponded to the vitamins found in a 5 g·L^−1^ yeast extract containing medium (Table 1).

The cellular extracts are *Saccharomyces cerevisiae* and *Escherichia coli* autolysis samples: *Saccharomyces cerevisiae* extract was obtained from a 4 h culture of the strain CEN.PK 113-7D in YPD medium (yeast extract 10 g·L^−1^, bacto-peptone 20 g·L^−1^ and glucose 100 g·L^−1^) at 30 °C. After one centrifugation step 10 min at 4000 g (Centrifuge 5810 R, Eppendorf, Hamburg, Germany), the pellet was suspended in 0.1 M pH 5 citrate buffer. Autolysis was performed at 52 °C during 72 h on a rotary shaker at 140 rpm [22]. For *Escherichia coli* extract, a 12 h growth of *Escherichia coli* K12 at 37 °C in LB medium (tryptone 10 g·L^−1^, yeast extract 5 g·L^−1^, NaCl 10 g·L^−1^ and glucose 10 g·L^−1^) was centrifuged 10 min at 4000 g. Cells pelleted were resuspended in milliQ water (18.2 MΩ cm, Millipore, Darmstadt, Germany) and 0.5 M sodium acetate buffer (pH 6.5) 1:1 (v/v). Autolysis was obtained within 48 h of incubation at 37 °C without shaking [23].

**Table 1:** Vitamin supplementation in defined medium.

Vitamins	Concentration (mg·L^−1^)
Thiamine	0.105
Riboflavine	0.625
Pyridoxine	0.12
Nicotinic acid	3
Panthotenic acid	0.525
Folic acid	0.03
Choline	7.5
Biotine	0.02

*Deinococcus geothermalis* DSM-11302 extract was prepared from a culture in CMG, 24 h at 45 °C. The medium was centrifuged 15 minutes at 4000 g, the cells were rinsed three times with physiological solution (NaCl 9 g·L^−1^) and resuspended in a buffer solution (NaCl 9 g·L^−1^ + MOPS 40 mM). The cells were disrupted by mechanical lysis using a French press (220 V, 50 Hz, FA-078A-E Thermo Electron Corporation, Waltham, Massachusetts, MA, USA): the sample was submitted 3 times to the desired pressure (10,000 psi).

These experiments were performed using DM medium without glucose for carbon source assimilation tests and with DMG for others tested conditions.

#### 2.1.4. Determination of the Respiratory Type of *Deinococcus geothermalis* DSM-11302

The growth was tested in an environment with or without oxygen. A culture of *D. geothermalis* DSM-11302 was transferred into YPDA tubes (YPD medium broth supplemented with agar 6 g·L^−1^) by stabbing with a sterile inoculation loop. In Petri dishes YPDA was inoculated by spread plate technique, 0.1 mL of bacterial suspension was spread over the surface on the agar medium, or by streaking with an inoculation loop. To test the growth in the absence of oxygen, the pouring plate technique was used. A sterile empty Petri dish was inoculated with 0.1 mL of bacterial suspension and the YPDA medium was poured into the Petri dish. A second layer of agar medium was poured in the dish after the solidification of the first one. Tubes and Petri dishes were incubated at 45 °C during 7 days in ambient air.

#### 2.1.5. Culture in Bioreactor

The batch experiment was performed in a 1.6 L bioreactor BBraun Biostat Bplus (Sartorius, Göttingen, Germany) of 1 L working volume. The reactor was instrumented with dissolved oxygen probe (OxyFerm FDA 160, Hamilton, Bonaduz, Switzerland), pH probe (Fermprobe Broadley-James Co., Santa Ana, CA, USA), temperature and pressure sensors. The mixing system consists of four baffles and 2 six-bladed Rushton impellers. Stirring and airflow were regulated to avoid oxygen limiting conditions. The temperature was set at 45 °C with a water-filled jacket. The pH was kept at 6.8 with addition of 14% (v/v) ammonia solution or 2 M ortho-phosphoric acid solution. The antifoam agent struktol J673 (Struktol, Hamburg, Germany) was used in case of foam formation during the fermentation. On-line acquisition and regulation were done using MFCS/win 2.1 software package (Sartorius, Göttingen, Germany). Throughout the experiment, samples were harvested and stored at −20 °C.

The composition of inlet and exhaust gas of the reactor was measured with a gas analyser Innova 1313 (LumaSense Technologies, Ballerup, Denmark), by infrared-acoustic spectroscopy for carbon dioxide and by magneto-acoustic spectroscopy for oxygen. These measurements, with mass balance Equation (1), allowed calculating oxygen uptake and carbon dioxide production rates and respiratory quotient (RQ). RQ is defined by the ratio between carbon dioxide production rate (*r*_CO2_) and oxygen uptake rate (*r*_O2_); its value depends on degrees of reduction of the carbon source (γ_S_), the final electron acceptor (γ_O2_) and the biomass (γ_X_).

(1) $$RQ=\left|\frac{r_{CO_{2}}}{r_{O_{2}}}\right|=\left|\gamma_{O_{2}}\times \frac{1-Y_{S,X}}{\gamma_{S}-\gamma_{X}\times Y_{S,X}}\right|$$

#### 2.1.6. Chemical Products

Glucose, fructose, salts, ammonia and oligo-elements were obtained from Prolabo (VWR International, Fontenay-sous-Bois, France), sulphuric acid from Fisher (Fisher Scientific, Illkirch, France) and yeast extract and bacto-peptone from BD Diagnostics (Le Pont de Claix, France). ZnSO_4_ was provided from Carlo Erba (Val de Reuil, France) and MgCl_2_, vitamins, amino acids and the MOPS were provided from Sigma-Aldrich (Lyon, France). All products were of the highest analytical grade available. 

### 2.2. Analytical Methods

#### 2.2.1. Biomass Analysis

Biomass concentration was determined by optical density (OD_600 nm_) measurement at 600 nm (spectrophotometer Libra S4, Biochrom, Cambridge, UK) with a 2 mm absorption cell (Hellma, Müllheim, Germany) and cell dry weight measurements. Cell dry weight was estimated by filtration on polyamide membrane (Sartolon 0.2 µm-Sartorius^®^, Sartorius, Göttingen, Germany) and drying to a constant weight for 48 h, at 60 °C under 200 mmHg in a vacuum oven (Heraeus, Hanau, Germany).

#### 2.2.2. Supernatant Analysis

During the fermentation, culture supernatant was obtained by centrifuging (MiniSpin Eppendorf, Hamburg, Germany) fermentation broth samples in Eppendorf tubes at 12,000 g for 3 min. The glucose concentration in supernatant was measured with a YSI 2700 glucose analyzer (Yellow Springs Instruments, Yellow Springs, OH, USA). Glucose and organic acid concentrations of filtered supernatants (Minisart filters 0.2 µm size pore, Sartorius, Göttingen, Germany) were determined by HPLC (Ultimate 3000, Dionex, Thermo Fisher Scientific, Courtaboeuf, France) using an Aminex HPX-87H+ column (Bio-Rad Life Science, Marnes-la-Coquette, France) under the following conditions: 50 °C, 5 mM H_2_SO_4_ as mobile phase at a flow rate of 0.5 mL·min^−1^ and detection with a refractometer and an UV detector at 210 nm. The samples were previously diluted in deionised water. External standards (0.2 to 5 g·L^−1^) were used for compound identification and quantification. Chromatograms were analyzed with Chromoleon software 6.80 (Thermo Fisher Scientific, Courtaboeuf, France). For the flask experiments, the glucose uptake and the biomass yield were calculated at the end of the growth phase.

## 3. Results and Discussion

### 3.1. Comparison of the Growth of Deinococcus geothermalis Strains DSM-11300, DSM-11301 and DSM-11302

The growth of three strains of *D. geothermalis*, DSM-11300, DSM-11301 and DSM-11302, was compared in CMG, TH162 medium and two defined media: Holland medium and DMG. The results of growth are shown in Table 2.

**Table 2:** Number of generations and percentage of glucose uptake after 24 h and 48 h of growth of *Deinococcus geothermalis* (Deutsche Sammlung von Mikroorganismen, DSM) DSM-11300, DSM-11301 and DSM-11302 in Defined Medium Glucose (DMG), Complex Medium Glucose (CMG), *Thermus* 162 (TH162) medium and Holland medium.

Strains		*Deinococcus geothermalis* DSM 11300				*Deinococcus geothermalis* DSM 11301				*Deinococcus geothermalis* DSM 11302			
Media		DMG	CMG	TH162	Holland	DMG	CMG	TH162	Holland	DMG	CMG	TH162	Holland
24 h	Number of generations	1.3	3.7	1.4	0.1	1.6	3.7	1.6	0.3	2.8	3.7	1.6	0.4
	Glucose uptake (%)	16	75	/	/	41	86	/	/	55	94	/	/
48 h	Number of generations	1.6	3.7	1.4	0.1	1.6	3.7	1.6	0.3	3.2	4.1	1.6	0.4
	Glucose uptake (%)	24	100	/	/	81	100	/	/	89	100	/	/

No growth was observed in the medium described by Holland *et al.* [17] and 1.4–1.6 generations were obtained in TH162 medium. The best growth was obtained in the complex medium CMG for the three strains (3.7–4.1 generations). The strain *D. geothermalis* DSM-11300 experienced poor growth on DMG: only 1.6 generations were obtained in 48 h. However, with the strain DSM-11302, more than three generations were obtained in 48 h. In the two defined media and TH162 medium, flocculation of the strain *D. geothermalis* DSM-11301 was observed despite the weak growth.

The results showed that the TH162 medium and the Holland medium were not adapted for the growth of these strains of *D. geothermalis*. The tested conditions induced stress toward the strain DSM-11301 which formed pellets. Although the four strains belong to the same species, they did not have the same behaviour in the tested media. *D. geothermalis* DSM-11300 and DSM-11302 have both been isolated from hot springs, but not in the same location. The strain DSM-11300 has been recovered from water samples collected at the Termi di Agnano in Italy, whereas the strain DSM-11302 has been isolated at São Pedro do Sul in Portugal [2]. Environment varies with geographical locations, so it could explain why *D. geothermalis* strains had different physiological properties. Finally, the maximum biomass concentration was obtained with DSM-11302, in both media CMG and DMG. According to these results, it seemed that *D. geothermalis* DSM-11302 is less fastidious and could produce more biomass in DMG than the others strains of *D. geothermalis*.

### 3.2. Effect of the Temperature on the Growth of *Deinococcus geothermalis* DSM-11302 and Respiratory Type

**Figure 1:**
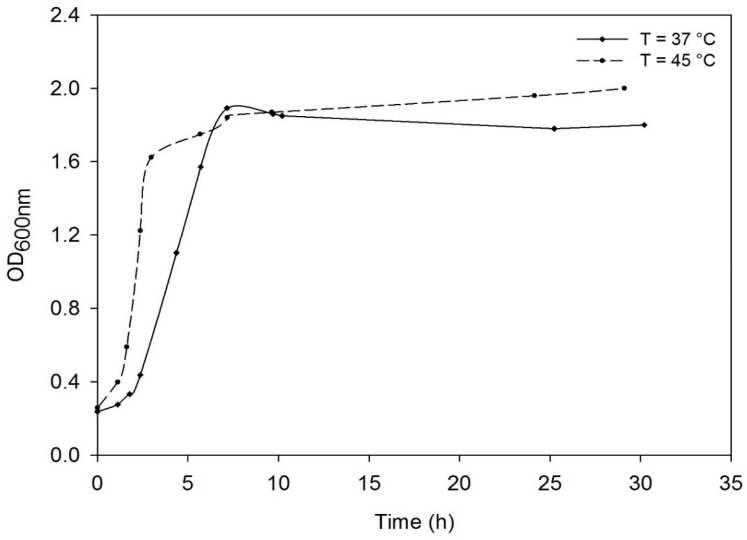
Comparison of growth curve of *Deinococcus geothermalis* (Deutsche Sammlung von Mikroorganismen, DSM) DSM-11302 at 37 °C and 45 °C in Complex Medium Glucose.

The growth of *D. geothermalis* DSM-11302 was compared in CMG at 37 °C and 45 °C (Figure 1).

At 37 °C, the growth rate was 0.47 h^−1^ and the maximal optical density at 600 nm (OD_600 nm_) 1.8 whereas at 45 °C the growth rate was 0.79 h^−1^ and the maximal OD_600 nm_ 2. It is important to note that temperature positively affected growth rate, but had no effect on the maximal biomass concentration. 

As oxygen requirement for growth relates to the energy metabolism of the microorganism, this study included the investigation of the respiratory type of *D. geothermalis*. After seven days of incubation, the growth of *D. geothermalis* occurred only when oxygen was available, at the top of the YPDA tubes and at the top of the agar plates. The oxygen is used as electron acceptor; this microorganism is strictly aerobic which is not surprising because *D. geothermalis* DSM-11302 is an aerobic bacterium firstly isolated from hot springs [2]. This result is in accordance with Liedert and co-workers who described that in rich culture conditions *D. geothermalis* aerobic metabolism is active [24]. These authors have proposed that *D. geothermalis* grew in fully-oxygenated conditions but suffered from oxidative stress; the response of this kind of stress being a modification of its metabolism to produce reactive oxygen species (ROS)-combating metabolites. Whether in tubes or in Petri dishes, *D. geothermalis* DSM-11302 cultivated in rich environment formed orange-pigmented colonies. This color is due to carotenoids like deinoxanthin that could be responsible for the antioxidant activity of these bacteria submitted to ROS [5,25]. 

### 3.3. Effect of Yeast Extract Concentration

**Figure 2:**
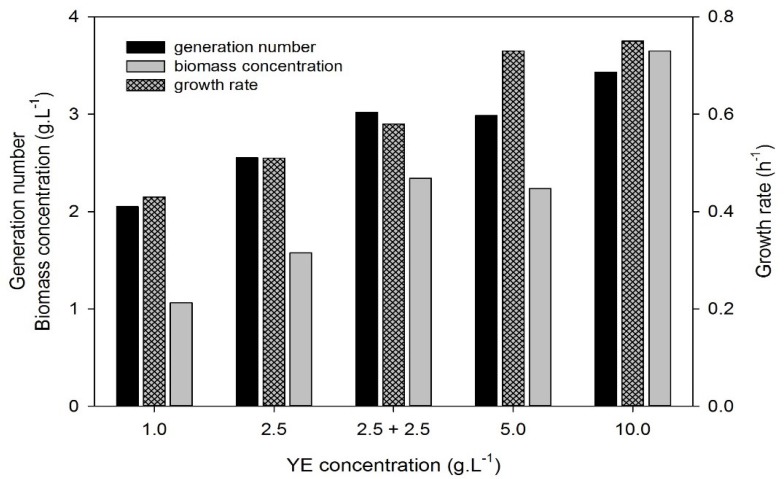
Effect of yeast extract concentration on the culture of *Deinococcus geothermalis* DSM-11302 in Complex Medium Glucose in terms of number of generations, biomass concentration and growth rate. 2.5 + 2.5 means that 2.5 g·L^−1^ yeast extract (YE) were in the initial medium and a supplementary pulse of 2.5 g·L^−1^ YE was made when the culture reached the stationary phase.

The addition of yeast extract varying from 1 to 10 g·L^−1^ was tested in Erlenmeyer flask experiments in order to study the correlation between yeast extract concentration and the final biomass concentration of *D. geothermalis*. The results are shown in Figure 2.

The growth rate, the number of generations and the percentage of glucose uptake increased with yeast extract concentration. The maximal growth rate was obtained with a minimum yeast extract concentration of 5 g·L^−1^. This increase of the growth rate could be explained by the presence of one or many elements in yeast extract acting as catalysts with a sufficient amount in the medium when 5 g·L^-1^ of yeast extract were added. The maximum number of generations, 3.4, was obtained with 10 g·L^−1^ of yeast extract; doubling the concentration of yeast extract from 5 to 10 g·L^−1^ did not result in a significant increase of the number of generations. With yeast extract concentrations higher than 5 g·L^−1^, it was possible that the factor limiting the growth in the flask culture was the dissolved oxygen concentration.

The biomass concentration of *D. geothermalis* DSM-11302 was proportional to yeast extract concentration in the tested conditions. It confirmed that yeast extract provided also constitutive elements of the biomass. Even if the glucose uptake increased with the concentration of yeast extract, the compounds used as carbon source to produce biomass were mainly provided by yeast extract. An increase of the final pH value was observed, probably as a consequence of the catabolism and the lysis of peptides and proteins resulting in ammonia liberation in the culture medium. It has already been reported for a *Deinococcaceae* strain, that in a rich medium, the biomass formation is mainly supported by protein-containing components in the medium, tryptone and yeast extract [16,26]. Even if the glucose is consumed, *D. radiodurans* does not assimilate glucose efficiently and its role in biomass formation is not significant [26]. Our results suggest that it is the case for *D. geothermalis* as well; this organism is mainly proteolytic.

Two modes of yeast extract supply were also studied; in one case, 5 g·L^−1^ was initially added in the culture medium and in the other case, two times 2.5 g·L^−1^ was sequentially added: at the beginning of the culture and when cells entered the stationary phase. The same final OD_600 nm_ and number of generations were reached. Moreover, as previously described, when 5 g·L^−1^ of yeast extract was added in the medium at the beginning of the culture, a 26% increase of growth rate was observed compared to the second supply mode. So, sequenced yeast extract supplies had no impact on final biomass concentration and number of generation but significant impact on the kinetics; yeast extract brings both constitutive and catalytic elements. Further analysis of the nutritional requirements of *Deinococcus geothermalis* have been performed: a respirometry-based method has been developed to study the major growth supporting components, and the results have revealed that the whole yeast extract in the medium is essential to obtain a non-limiting growth [18].

### 3.4. Effect of Method of Strain Preservation, Frozen Storage Period, Type of Strain Revivification and Variability between CFU on Petri Dishes

The influence of the methods of preservation and revivification of *D. geothermalis* DSM-11302 on the growth rate and/or on the final biomass concentration was checked in CMG or in DMG (Table 3 and Table 4).

When *D. geothermalis* was grown in CMG, the growth rate reached 0.75 ± 0.02 h^−1^ and the number of generations was 2.5 ± 0.2. In DMG, the growth rate value varied from 0.15 h^−1^ to 0.17 h^−1^ and the number of generations was 2.6 on average. The growth performances were independent of the strain preservation and revivification methods and the duration of the storage in the conditions tested. Furthermore, these inoculum preparation steps had no significant effect on the biomass yield and the glucose uptake.

### 3.5. Influence of Inoculation Size and Duration of Incubation Phase of Pre-Culture

The comparison of the growth of *D. geothermalis* DSM-11302 was made with precultures incubated from 4 to 24 h. Two inoculation sizes, 10% and 20% (v/v) with a 12 h preculture, were tested too. The main growth parameters determined are summarized in Table 5.

The growth rate reached 0.14 h^−^^1^ and 0.20 g_x_·g_Glc_^−1^ were obtained with the two tested inoculation sizes. With an inoculum size equalled 10%, 2.9 generations were obtained, and with an inoculum size of 20% the number of generations was 2.2. Doubling the inoculation size increased neither the growth rate nor the biomass concentration.

**Table 3:** Effect of the strain preservation methods, the frozen storage period, the type of strain revivification and the variability between colony forming unit on Petri dishes on the culture of *Deinococcus geothermalis* DSM-11302 in CMG.

Medium	CMG						
Preservation method	Freeze dried cells		Glycerol stocks (−80 °C)				
Storage period (months)	–		20	12			
Revivification	Petri dishes culture	Direct tube inoculation	Petri dishes culture	Petri dishes culture	Petri dishes culture	Petri dishes culture	Direct tube inoculation
Initial OD_600 nm_	0.282	0.334	0.286	0.270	0.270	0.338	0.324
Final OD_600 nm_	1.58	1.77	1.63	1.72	1.72	1.62	1.72
Number of generations	2.5	2.4	2.5	2.7	2.7	2.3	2.4
µ (h^−1^)	0.76	0.75	0.75	0.76	0.76	0.76	0.71
Glucose uptake (%)	44	42	41	50	60	40	42

**Table 4:** Effect of the strain preservation methods, the frozen storage period, the type of strain revivification and the variability between colony forming unit on Petri dishes on the culture of *Deinococcus geothermalis* DSM-11302 in DMG.

Medium	DMG						
Preservation method	Freeze dried cells		Glycerol stocks (–80 °C)				
Storage period (months)	–		20	12			
Revivification	Petri dishes culture	Direct tube inoculation	Petri dishes culture	Petri dishes culture	Petri dishes culture	Petri dishes culture	Direct tube inoculation
Initial OD_600 nm_	0.232	0.224	0.216	0.233	0.246	0.22	0.218
Final OD_600 nm_	1.37	1.37	1.34	1.35	1.36	1.36	1.52
Number of generations	2.6	2.6	2.6	2.5	2.5	2.6	2.8
µ (h^−1^)	0.17	0.17	0.16	0.15	0.16	0.15	0.15
Glucose uptake (%)	38	36	42	50	26	40	51
Y_S,X_ (g_x_·g_Glc_^−1^)	0.40	0.44	0.36	0.31	0.59	0.41	0.35

With the precultures aged from 4 to 12 h, the exponential growth rates were higher than 0.11 h^−^^1^, whereas with older precultures a slightly lower growth rate was obtained, 0.09 h^−^^1^. The highest yield of biomass production on glucose, 0.31 g_x_·g_Glc_^−1^, was obtained with the preculture of 8 h but the age of the preculture did not have a significant influence on the number of generations obtained during the culture. A preculture of 4–12 h used at an inoculum size of 10% was sufficient to achieve the maximal biomass concentration and growth rate. Nevertheless, a preculture older than 12 h was able to grow in fresh medium. No morphological differences were highlighted with microscopic observations. Even after an incubation of 24 h, no lysis of the cells was observed; *D. geothermalis* DSM-11302 cultivated in rich medium remained very resistant. This result is in accordance with previously published works which have shown the robustness of *Deinococcus* species against physical and chemical stresses [1,2,27].

**Table 5:** Effect of the inoculation size and the duration of incubation of the preculture on the results of growth of *Deinococcus geothermalis* DSM-11302 in DMG.

Age of Pre-Culture (h)	4	8	12		16	20	24
Inoculation size (%)	10	10	10	20	10	10	10
Initial OD_600 nm_	0.201	0.270	0.213	0.439	0.203	0.231	0.260
Final OD_600 nm_	1.72	1.79	1.61	2.00	1.41	1.51	1.57
Number of generations	3.0	2.7	2.9	2.2	2.7	2.7	2.6
µ (h^−1^)	0.17	0.11	0.14	0.14	0.09	0.09	0.08
Glucose uptake (%)	45	42	40	55	37	28	40
Y_S,X_ (g_x_·g_Glc_^−1^)	0.26	0.31	0.19	0.21	0.22	0.29	0.23

### 3.6. Influence of Successive Subcultures, Dilution of The Residual Yeast Extract in the Medium and Cell Washing before Inoculation on the Growth of Deinococcus geothermalis DSM-11302 in DMG

To study the influence of the residual yeast extract, the pellet was washed one to three times with physiological saline solution before inoculation. The results are shown in Table 6.

In the defined medium DMG, the number of cell washing steps had no significant influence on the growth of *D. geothermalis* DSM-11302, in terms of growth rate, number of generations and biomass yield. With one cell washing, the residual medium in the pellet was 1:5000 diluted and with three steps of cell washing, it was 1:1.25 × 10^9^ diluted. One step of cell washing is enough to remove the medium containing yeast extract in the pellet. The growth observed in DMG was not due to a residual part of yeast extract transfer during the inoculation step.

One to three successive precultures in complex media were tested; there was no significant influence on the number of generations in DMG but it caused a decrease of the growth rate, 0.13 h^−1^ with one preculture compared to 0.06 h^−1^ with three successive precultures (Table 6). So, when the number of generations in the precultures increased, the capacity of growth of the microorganism, according to kinetic results (growth rate), decreased.

### 3.7. Growth of Deinococcus geothermalis DSM-11302 in Defined Media on Carbon Sources and Complex Nutrients

To choose a mineral medium suitable for the growth of *D. geothermalis* DSM-11302, the carbon source supporting the best growth was determined (Table 7): the highest number of generations, 3.1, was obtained with glucose, trehalose and sucrose. With mannose, fructose and maltose, less biomass was produced; the number of generations was between 2.5 and 2.9. Only one generation was obtained in the medium with galactose as carbon source and no growth was observed on carboxymethylcellulose or lactose, although lactose has been previously described as a carbon source suitable for *Deinococcus geothermalis* DSM-11300 growth [2].

The results observed with trehalose, maltose, mannose, galactose, sucrose, fructose and glucose are in accordance with data published on the growth of *D. geothermalis* DSM-11300 [2,14]. In the defined medium used in this study, glucose is a carbon source well-adapted for the growth of *D. geothermalis* DSM-11302.

Several published results have reported that *Deinococcaceae* are proteolytic microorganisms and prefer other carbon sources than glucose in yeast extract and tryptone, like amino acids [2,28]. With a *D. geothermalis* strain, the best growth performances described to date have been obtained using a glucose-yeast extract co-substrate fed-batch culture [15]. So, five solutions were supplemented to the basal defined medium: a solution containing yeast extract vitamins, a casamino acid vitamin-free solution providing all the essential amino acids except tryptophan, and three cellular extracts; the results are shown in Table 8.

The growth results, in terms of number of generations and yields, were not better when the cellular extracts were used as growth factors. A *D. geothermalis* extract cannot serve as a starter for a new culture of *D. geothermalis*. In addition, the stimulating effect of yeast extract is neither due to its vitamins, nor to the amino-acids. When compared to the defined medium composition, the supplementations tested could not improve the growth performances of *D. geothermalis* DSM-11302. 

It was possible to obtain three generations and 0.23 g_x_·g_Glc_^−1^ with this mineral medium containing glucose as a carbon source.

The strain was then cultivated in a bioreactor with the mineral medium DM and glucose as substrate, using a batch culture mode in order to quantitatively evaluate the growth behaviour on minimal medium in well-controlled conditions and to determine the physiological characteristic parameters (kinetic parameters, respiratory quotient and yield) of *D. geothermalis* DSM-11302 type strain. 

**Table 6:** Effect of successive subcultures and cell washing steps before inoculation on the results of growth of *Deinococcus geothermalis* DSM-11302 in DMG.

Precultures nb	1	2	3	1	1	1
Cell washing nb	1	1	1	1	2	3
Dilution of residual yeast extract	2.10^−4^	2.10^−4^	2.10^−4^	2.10^−4^	4.10^−7^	8.10^−10^
Initial OD_600 nm_	0.192	0.245	0.233	0.292	0.285	0.239
Final OD_600 nm_	1.76	1.84	1.90	1.70	1.66	1.62
Number of generations	3.2	2.9	3.0	2.5	2.5	2.8
µ (h^−1^)	0.13	0.10	0.06	0.13	0.13	0.13
Glucose uptake (%)	63	67	45	50	53	44
Y_S,X_ (g_x_·g_Glc_^−1^)	0.14	0.15	0.25	0.20	0.18	0.25

**Table 7:** Effect of nine carbon sources on the results of growth of *Deinococcus geothermalis* DSM-11302 in DMG.

Carbon Sources	CMC (Carboxy Methyl Cellulose)	Mannose	Trehalose	Galactose	Fructose	Maltose	Lactose	Sucrose	Glucose
Initial OD_600 nm_	0.269	0.257	0.245	0.296	0.272	0.261	0.248	0.240	0.242
Final OD_600 nm_	0.222	1.880	2.120	0.690	1.540	1.990	0.218	1.990	2.000
Number of generations	0	2.9	3.1	1.2	2.5	2.9	0	3.1	3.1

**Table 8:** Effect of five complex sources on the results of growth of *Deinococcus geothermalis* DSM-11302 in DMG.

Complex Sources	YE (Yeast Extract) Vitamins	Casamino Acids	*D. geothermalis* Extract	*S. cerevisiae* Extract	*E. coli* extract	None
Initial OD_600 nm_	0.242	0.248	0.288	0.301	0.230	0.246
Final OD_600 nm_	1.840	1.650	1.850	2.000	1.760	1.860
Number of generations	2.9	2.7	2.7	2.7	2.9	2.9
Glucose uptake (%)	100	90	100	100	100	94
Y _S,X_ (g_x_·g_Glc_^−1^)	0.23	0.21	0.23	0.23	0.23	0.23

### 3.8. Quantification of Deinococcus geothermalis DSM-11302 Growth on a Mineral Medium

**Figure 3:**
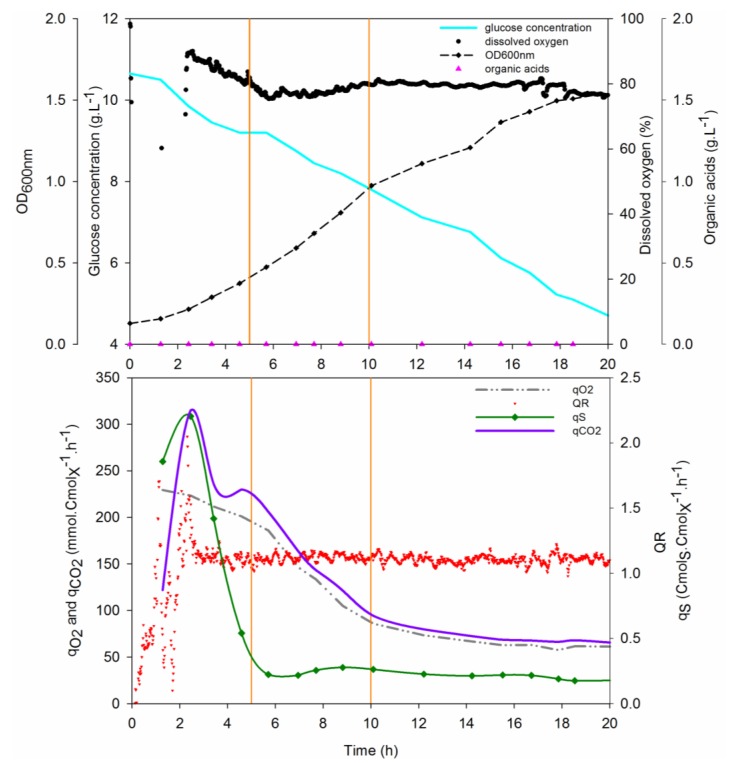
Time course evolution of the optical density OD_600 nm_, the glucose concentration, the dissolved oxygen value, the acidic by-products concentration, the respiratory quotient (QR) and the specific oxygen uptake, carbon dioxide production and glucose uptake rates during batch culture of *Deinococcus geothermalis* DSM-11302 (vertical bars represent the three phases of growth).

The growth of *D. geothermalis* DSM-11302 in a bioreactor using defined medium with 10 g·L^−1^ of glucose is shown on Figure 3.

The growth can be divided in three phases based on changes in growth and oxygen uptake:

Phase I: the culture started with an exponential growth during 5 h; 1.2 generations were obtained at a maximum growth rate of 0.28 h^−1^ and the oxygen uptake (*r*_O2_) and carbon dioxide production (*r*_CO2_) rates reached, respectively, 3.3 and 3.5 mmol·L^−1^·h^−1^. During this exponential growth, the specific oxygen uptake rate (*q*_O2_) was on average 216 mmol Cmol_X_^−1^·h^−1^ and the specific carbon dioxide production rate (*q*_CO2_) was 226 mmol.Cmol_X_^−1^·h^−1^. QR value was 1.17 during this phase and the glucose was consumed at a maximum rate equal to 2.2 Cmol Cmol_X_^−1^·h^−1^.

Phase II: five h after the beginning of the culture, the dynamic of growth decreased. Between 5 and 6 h of culture, the oxygen uptake slowed down; after that decrease, the p_O2_ value stabilized at between 75% and 80%. The oxygen uptake rate (*r*_O2_) held around 3.4 mmol_O2_·L^−1^·h^−1^ whereas during this second five hour growth phase, a biomass corresponding to 1.5 generations was produced. So, a decrease of 40% of the oxygen uptake and carbon dioxide production rates was observed. The growth rate was on average 0.16 h^−1^, q_Glc_ steady state value was 0.22 Cmol·Cmol_X_^−1^·h^−1^ and the QR 1.12.

Phase III: a linear growth phase of 8 h (0.04 h^−1^) was then observed, with no change in the physiological behaviour of the microorganism; the substrate consumption rate was maintained at 0.22 Cmol·Cmol_X_^−1^·h^−1^ while *q*_O2_ and *q*_CO2_ stabilized at 67 mmol_O2_·Cmol_X_^−1^·h^−1^ for *q*_O2_ and 73 mmol_CO2_·Cmol_X_^−1^·h^−1^ for *q*_CO2_. The oxygen uptake and carbon dioxide production rates were 3.3 and 3.6 mmol·L^−1^·h^−1^, respectively. The steady state value of *p*_O2_ was 80% and the QR value was 1.12. During this phase, the growth continued but the oxygen uptake stayed steady even if glucose was still available in the medium. This behaviour could result from a nutritional starvation in the medium. At the end of the culture, nutrients were added in the medium to restore the growth dynamic but no effects were highlighted. The kinetic parameters quantified during this culture are summarized in Table 9.

**Table 9:** Kinetic growth parameters of *Deinococcus geothermalis* DSM-11302 under controlled nutritional and culture conditions.

Physiological Parameters	Phase I	Phase II	Phase III
Growth rate (h^−1^)	0.28	0.16	0.04
Number of generations	1.2	1.5	–
*q*_O2_ (mmol_O2_·Cmol_X_^−1^·h^−1^)	216	186 ↘ 105	67
*q*_CO2_ (mmol_CO2_·Cmol_X_^−1^·h^−1^)	226	206 ↘ 121	73
*q*_Glc_ (Cmol_Glc_·Cmol_X_^−1^·h^−1^)	2.2	0.22	0.22
QR	1.7	1.12	1.12

The culture began with an exponential growth phase with a growth rate of 0.28 h^−1^, then it decreased. The bioreactor culture allowed doubling the growth rate on the mineral medium, e*.*g., 0.15 h^−1^ on average in Erlenmeyer flasks. The glucose uptake rate varied from 2.2 to 0.22 Cmol_Glc_·Cmol_X_^−1^·h^−1^ during the growth. In the whole culture, the growth rate was on average 0.13 h^−1^ and 5.55 g of glucose was taken up and 1.38 g of dry cell weigh produced. In the mineral medium DM with glucose as carbon source, the yield reached 0.25 g_x_·g_Glc_^−1^ or 0.30 Cmol_X_·Cmol_Glc_^−1^. The value of 2.7 generations is close to the values reached in Erlenmeyer flasks (between 2.6 and 3 generations). Furthermore, growth of *D. geothermalis* DSM-11302 on excess glucose under aerobic conditions did not cause the formation of acidic by-products (such as formate, lactate, malate, succinate, pyruvate, acetate, citrate and gluconate); no overflow metabolism was revealed under these conditions.

## 4. Conclusions

The growth of three type strains of *Deinococcus geothermalis* was compared in complex and defined media and the results highlighted the strain DSM-11302 as the best candidate for culture in CMG and DMG. This species was found to be less fastidious than the other ones. The variability of physiological behaviour between these strains could be related to the geographical locations where they have been isolated; differences of biochemical properties have already been described by Ferreira *et al.*, in carbon source assimilation studies [2].

This study confirmed that *D. geothermalis* DSM-11302 is an aerobic bacterium, reaching a growth rate equal to 0.17 h^−1^ at 45 °C in the defined medium DMG. However, yeast extract was necessary to achieve a non-limiting growth at a maximal growth rate of 0.76 h^−1^. Furthermore, the final biomass concentration and the growth rate were correlated with yeast extract concentration. Few hypotheses could explain the positive effect of yeast extract on the growth: yeast extract brings constitutive elements to the cells, it could provide growth factors like vitamins or enzyme cofactors and it could have a catalytic effect too. Even if data in the literature have highlighted a significant role of Mn and Mg for the growth of *D. radiodurans* [16,17,26], a trace element supplemented by yeast extract, our experiments did not confirm these observations for *D. geothermalis* [18]. This is not surprising since the mesophilic and the thermophilic strains are described to exhibit different metabolic capacities. The results of Brim and co-workers have revealed that *D. geothermalis* and *D. radiodurans* have distinct substrate utilization and behaviour [14]. The thermophilic species is able to grow on fructose and several Embden-Meyerhof-Parnas substrates and use ammonium sulphate as a nitrogen source; *D. geothermalis* has been described as independent of amino acids for its growth in the absence of irradiation. In contrast, *D. radiodurans* is less robust since its growth on fructose requires the presence of amino acids.

The results of growth, in terms of maximal biomass concentration, number of generations and biomass yield, were not affected by the methods of strain preservation and revivification. *D. geothermalis* DSM-11302 is a very robust microorganism capable of surviving 24 h of preculture with minimal consequences for its growth properties. Moreover, it is not affected by the cell washing steps before the inoculation. The growth of *D. geothermalis* DSM-11302 in DMG was not explained by yeast extract transfer from the complex medium to the defined medium during the inoculation step. However, the results revealed that the accumulation of cell divisions in precultures resulted in a progressive decrease of the growth rate of *D. geothermalis* DSM-11302. 

Using well-controlled batch culture, it was possible to obtain an exponential growth of *D. geothermalis* DSM-11302 on glucose-mineral medium. Cells grew exponentially for 4 h at 0.28 h^−1^ maximal specific growth rate. Then, in spite of glucose in the medium, the growth rate decreased at 0.16 h^−1^ and then 0.04 h^−1^. Physiological parameters of *D. geothermalis* have been characterized in these conditions: the oxygen uptake rate reached 3.3 mmol_O2_·L^−1^·h^−1^, corresponding to a specific value of 230 mmol_O2_·Cmol_X_^−1^·h^−1^, the QR value was 1.17 during the exponential growth phase and the glucose uptake rate was on average 0.57 Cmol_Glc_·Cmol_X_^−1^·h^−1^. In this work, 5.6 g of glucose was consumed in the culture medium to produce 1.4 g_DryCellWeight_ of biomass, which corresponds to 0.3 Cmol_X_·Cmol_Glc_^−1^. No overflow metabolism was detected under these cultivation conditions.

This work reports the first description of an exponential growth of a *Deinococcus* strain in a bioreactor on a mineral medium, with the quantitative characterization of kinetic parameters (growth rates, substrate uptake rate, respiration rates, respiratory quotient), data which are crucial for quantitative comparative studies. This led to a better understanding of the metabolism of *D. geothermalis* under controlled nutritional and environmental growth conditions. Such data provide knowledge on quantitative physiology of *Deinococcaceae* and are important for quantitative comparisons of the strain behaviours with published data.
